# Can zinc pollution promote adaptive evolution in plants? Insights from a one-generation selection experiment

**DOI:** 10.1093/jxb/ery327

**Published:** 2018-09-12

**Authors:** Julien Nowak, Hélène Frérot, Nathalie Faure, Cédric Glorieux, Clarisse Liné, Bertrand Pourrut, Maxime Pauwels

**Affiliations:** 1 Université de Lille, CNRS, UMR – Unité Evolution-Ecologie-Paléontologie, Lille, France; 2 ISA, Laboratoire Sols et Environnement, Lille Cedex, France

**Keywords:** Abiotic stress, anthropogenic habitats, experimental evolution, hyperaccumulation, fitness estimation, local adaptation, tolerance

## Abstract

Human activities generate environmental stresses that can lead plant populations to become extinct. Population survival would require the evolution of adaptive responses that increase tolerance to these stresses. Thus, in pseudometallophyte species that have colonized anthropogenic metalliferous habitats, the evolution of increased metal tolerance is expected in metallicolous populations. However, the mechanisms by which metal tolerance evolves remain unclear. In this study, parent populations were created from non-metallicolous families of *Noccaea caerulescens*. They were cultivated for one generation in mesocosms and under various levels of zinc (Zn) contamination to assess whether Zn in soil represents a selective pressure. Individual plant fitness estimates were used to create descendant populations, which were cultivated in controlled conditions with moderate Zn contamination to test for adaptive evolution in functional traits. The number of families showing high fitness estimates in mesocosms was progressively reduced with increasing Zn levels in soil, suggesting increasing selection for metal tolerance. In the next generation, adaptive evolution was suggested for some physiological and ecological traits in descendants of the most exposed populations, together with a significant decrease of Zn hyperaccumulation. Our results confirm experimentally that Zn alone can be a significant evolutionary pressure promoting adaptive divergence among populations.

## Teaser

Exposure to high levels of Zn generates variation in reproductive success among non-metallicolous individuals of *Noccaea caerulescens.* Descendant populations show slightly increased Zn tolerance and significantly decreased Zn hyperaccumulation.

## Introduction

Local adaptation is a major topic in evolutionary biology, especially in the current context of global change, because anthropogenic activities have generated environmental modifications in time and/or space (Grimm *et al.*, 2008). Modified environmental conditions might represent significant stresses for pre-existing populations of plants and other organisms, whose survival may rely on the capacity of individuals to either migrate or rapidly adapt their phenotype (Alberti *et al.*, 2017). There are several known examples of local adaptation after anthropogenic modifications. The first of these is the textbook example of industrial melanism in the British peppered moth (Majerus, 2009; van’t Hof *et al.*, 2016), and there is now an increasing number of examples from species evolving in urban areas (Johnson and Munshi-South, 2017). These examples suggest that local adaptation can occur rapidly. Most of the time, however, available data are not sufficient to support local adaptation, for a number of reasons. First, local adaptation is generally tested only indirectly, using correlations between environmental and phenotypic variations (Thompson *et al.*, 2016). Second, temporal data on phenotypic changes from ancestral to selected populations (Franks, 2011) are usually lacking, so that it is not possible to discuss the dynamics of evolution. Third, the absence of historical information does not allow us to exclude alternative scenarios for phenotypic evolution, so that any adaptive interpretation of currently observed patterns may be considered as a ‘just-so story’ (Olson and Arroyo-Santos, 2015).

In this context, experimental approaches allowing the testing of evolutionary hypotheses can be particularly helpful. Experimental evolution involves the development of experimental designs to test for the effects of evolutionary forces on the evolution of populations surveyed over time in either controlled or natural environments (Garland and Rose, 2009; Kawecki *et al.*, 2012). A particular type of experimental evolution involves selection experiments focusing on the role of selection in evolution (Fuller *et al.* 2005). A selection experiment combines experimental populations and environmental conditions to test whether an environmental parameter acts as a selective agent. Selection experiments are generally long-term experiments that follow hundreds to thousands of generations. This is, however, limited to organisms with (very) short generation times, such as *Escherichia coli* (Lenski, 2017) or *Drosophila melanogaster* (Graves *et al.*, 2017). In addition, some studies have revealed that adaptive changes can be observed over a smaller number of generations. These studies concerned the evolution of resistance to starvation in *D. melanogaster* (Kubrak *et al.*, 2017) or the reduction in the length of the life cycle in response to water stress in *Arabidopsis thaliana* (Brachi *et al.*, 2012). In some cases the effects of experimental selection can even be observed after one generation, for example, for the evolution of above-ground biomass or phenology in response to elevation-related stresses in hybrid populations of *Mimulus cardinalis* and *Mimulus lewisii* (Angert *et al.*, 2008).

Local adaptation to metalliferous environments represents a unique model to understand adaptive evolution in anthropogenic habitats. Anthropogenic metalliferous soils have elevated soil concentrations of trace metal elements (TMEs) such as zinc (Zn), cadmium (Cd), and lead (Pb). TMEs have a relatively low toxicity threshold and persist in the environment, and they can be considered important selective pressures for biological species (Sánchez, 2013). Thus, in pseudometallophyte plant species that colonize both metalliferous soils (metallicolous populations) and non-polluted soils (non-metallicolous populations), adaptive divergence between populations is expected. Accordingly, ecogeographic patterns showing phenotypic differentiation between metallicolous and non-metallicolous populations, with higher levels of tolerance to the TMEs to which they are exposed among metallicolous populations, is usually interpreted in adaptive terms, assuming that soil metals are the major selective pressures (Jiménez-Ambriz *et al.*, 2007; Dechamps *et al.*, 2008; Meyer *et al.*, 2009; Babst-Kostecka *et al.*, 2016).

Such an *a posteriori* interpretation of ecogeographic patterns can be challenged in various ways. There is no experimental evidence of the role of soil metal toxicity in adaptive divergence between metallicolous and non-metallicolous populations. On the contrary, recent data suggest that, besides metal pollution, other environmental factors that distinguish the ecology of metalliferous and non-metalliferous sites in anthropogenic landscapes may promote adaptive divergence among populations (Frérot *et al.*, 2018). Considering that metal tolerance can be defined as the ability to survive and reproduce on soils containing high concentrations of metals that are toxic for most plant species (Macnair, 1993), with no reference to any selective agents, any adaptation to a particular feature of metalliferous soils—even if it is not directly related to metal toxicity—could promote divergence in metal tolerance levels between metallicolous and non-metallicolous populations. This nuance may be even more relevant for pseudometallophytes showing species-wide quantitative variations in metal homeostasis mechanisms. In such species, non-metallicolous individuals showing basic physiological tolerance may be considered pre-adapted to internal metal toxicity (Meyer *et al.*, 2016), so that the evolution of increased tolerance to metalliferous soils could primarily require adaptation to other selective pressures. Finally, population genetic data suggest that demographic history may also play a role in between-population phenotypic divergence (Gonneau *et al.*, 2017). In some cases, trait values observed in metallicolous populations may not even be derived but rather may be ancestral (Babst-Kostecka *et al.*, 2018).

In this study, we developed a selection experiment on *Noccaea caerulescens*, a small Brassicaceae pseudometallophyte occurring on non-metalliferous, calamine (Zn- and Cd-enriched), and serpentine (Ni-enriched) soils. *N. caerulescens* is also known to hyperaccumulate Zn, Cd, and Ni. The species is a model system for the study of plant–soil interactions, particularly in metal-contaminated soils (Assunção *et al.*, 2003*b*; Milner and Kochian, 2008). Species-wide quantitative variation in metal tolerance and hyperaccumulation has been reported, and local adaptation to metal contamination has been assumed to account for phenotypic divergence between metallicolous and non-metallicolous populations (Meerts and van Isacker, 1997; Dechamps *et al.*, 2007; Gonneau *et al.*, 2014). Our first objective in this study was thus 2-fold. First, we wanted to investigate whether soil Zn pollution could represent a selective pressure promoting adaptive divergence among natural populations from standing genetic variation. To do so, we constructed several genetically homogeneous experimental populations from non-metallicolous progenies and subjected them to contaminated soil with different Zn doses in mesocosms for one generation. Plant performance was then estimated and compared, to assess whether selection could be assumed to be occurring. Second, we wanted to test whether one generation of selection from standing genetic variation could have modified the tolerance level of the descendant generation. To do this, performance estimates were used to create descendant populations. Descendant populations were exposed to Zn in controlled conditions and phenotyped in order to compare their tolerance levels. Tolerance tests were performed using a battery of morphological, physiological, and biochemical traits described in the literature.

## Materials and methods

### Design of parent experimental populations

In June 2013, maternal families were collected from three non-metallicolous populations of *N. caerulescens* in Luxembourg, at Winseler (WIN; 20 families), Wilwerwiltz (WIL; 15 families), and Lellingen (LE; 15 families) (Table 1). In September 2013, 15 seeds per family were sown in seedling trays containing peat-based compost, placed in a cold room at 4 °C for 1 week, and then transferred to a glasshouse for 6 weeks.

**Table 1.** Characteristics of the study sites

		Lellingen^*a*^	Wilwerwiltz^*b*^	Winseler^*b*^
**Geographic coordinates**		49°59′N, 6°00′E	49°57′N, 5°53′E	49°58′N, 5°59′E
**Soil pH**		5.7	5.9	5.8
**Total soil metal concentration (mg kg** ^−**1**^)	Zn	126 ± 4.3	139	164–274
	Cd	<1 ± 0.0	<2	0.7–4.3
				2.5–2.6
	Pb	48 ± 3.5	54	80–136
				122–168
	Ni	48 ± 2.6	42	66–157
				59–65

Soil samples were taken at a depth of 0–15 cm for study 1 and 0–10 cm for study 2. The concentration of metals is expressed as mg metal kg^−1^ soil.

^*a*^ Assunção *et al.*, 2003*a*.

^*b*^ Reeves *et al.*, 2001.

Available material was used to build four parental experimental populations (PPs). To be selected, a sown family had to have produced at least four seedlings to be represented once, and a multiple of four seedlings to be represented more than once, in each PP. Thus, each family was equally represented in the different PPs to homogenize the genetic composition of PPs. We selected as many family fulfilling these criteria as possible to maximize the genetic diversity within each PP. The final PPs were made up of 49 individuals from 23 families (13 from WIN, 10 from WIL, and 1 from LE; see Supplementary Table S1 at *JXB* online).

To confirm that PPs were genetically similar, all 196 individuals were genotyped using 14 microsatellite markers from the NcM1 and NcM3 multiplexes mentioned in Mousset *et al.* (2015). The extraction and genotyping protocols detailed in Mousset *et al.* (2015) were followed. Microsatellite markers Ncpm13 and Nc7b were removed from analyses because they showed no polymorphism in the natural populations we sampled (data not shown). Pairwise fixation index (*F*_ST_) values were calculated from microsatellite data using SPAGeDi 3 (Hardy and Vekemans, 2002).

### Transfer and cultivation of parent populations in outdoor mesocosms

In October 2013, PPs were transferred to distinct mesocosms. Mesocosms were made using square tubs designed from the lysimeter model proposed by Ruttens *et al.* (2006). Tubs had an area of 0.436 m^2^ enclosed by a 13 cm-wide buffer zone filled with compost to restrain border effects and create a thermic buffer (Supplementary Fig. S1). Tubs were filled with a mixture of 140 kg of peat- and clay-based compost and 70 kg of zeolite; this mixture was found to perform better than peat-based compost alone when following plants over their entire life cycle (unpublished data). Mesocosms were placed outdoors to allow plants to complete their life cycle under natural climatic conditions, in a 60 m^2^ external area; mesocosms were separated by at least 4 m (15 m maximum) to limit pollen transfer between them.

In three out of four mesocosms, different doses of zinc sulfate (ZnSO_4_·7H_2_O) were introduced in powder form to obtain soil contamination at concentrations of 500 mg kg^−1^ (439.73 g of ZnSO_4_·7H_2_O), 1000 mg kg^−1^ (879.46 g of ZnSO_4_, 7H_2_O), and 2000 mg kg^−1^ (1758.92 g of ZnSO_4_·7H_2_O). The fourth mesocosm was used as a non-contaminated control environment. In order to obtain homogeneous substrates, the mixtures were made in a cement mixer.

Each PP was named according to the level of Zn contamination to which it was exposed: PP_0_, PP_500_, PP_1000_, and PP_2000_. In late October 2013, seedlings were transferred into mesocosms at the center of cells of equal area arranged in a 7 × 7 grid (Supplementary Table S1). Plants were cultivated in their respective mesocosms until seeds were harvested in summer 2014.

### Estimation of individual plant fitness under Zn exposure in mesocosms

To assess the performance of plants in PPs under Zn exposure, and their ability to participate in forming the next generation, survival and several vegetative and reproductive traits were followed. The date of death and the corresponding phenological state were recorded for each plant. Several vegetative traits were measured at the emergence of the first flower bud. These comprised the plant surface (PS), calculated as the surface area of an ellipse from two orthogonal diameters of the rosette, the number of leaves (NL), and the mean leaf length (LL) and leaf width (LW), calculated from the three longest leaves. At the end of the life cycle, mature seeds were collected from every flowering plant that produced seeds. Plants were then harvested and several reproductive traits were measured. These included the number of flower stems (NFS), the length of the longest stem (maxLFS), the sum of the lengths of flower stems (sumLFS), the total number of non-aborted siliques (NS), and the mean length of siliques for the entire plant (meanLS), estimated from the length of five siliques per flower stem. The methodology for the measurement of meanLS was validated in a preliminary experiment conducted on 10 plants, on which we measured 100%, 75%, 50%, or 25% of siliques per stem, or 10 or five scattered siliques per stem. The best compromise between the effort of measurement and the absence of a significant difference from measuring 100% of siliques corresponded to measuring five siliques per stem (data not shown).

### Composition of descendant populations from fitness estimates

The contributions of maternal plants to the next generation, that is, the expected numbers of descendants, are related to their relative fitness (Violle *et al.*, 2007). They can be appropriately assessed by comparing reproductive outputs. In plants, reproductive output can be estimated from the seed set. However, owing to the large number of siliques and the initiation of fruit dehiscence and seed release by environmental factors such as rain and wind, it was not possible for us to exhaustively collect seeds and to measure seed set without risk of bias. Therefore, we used a proxy of seed number, called *W*_est_, calculated as the product of NS and meanLS (Brachi *et al.*, 2012; Roux *et al.*, 2016). Plant relative fitness (ω_est_) was then estimated by dividing the individual reproductive output *W*_est_ by the sum of reproductive outputs over all individuals from the same PP:

$$\omega_{est_{i}}=W_{est_{i}}/\Sigma_{i}^{49}W_{est_{i}}$$

where *i* is the *i*th plant among the 49 plants in the same PP.

After one generation of selection, four descendant populations (DPs) were expected from the four PPs, and were named according to the level of Zn contamination applied to the PPs: DP_0_, DP_500_, DP_1000_, and DP_2000._ The expected composition of the DPs was determined using the relative fitness of individual mother plants, ωesti, in the corresponding PPs to calculate an expected number of descendants (Fig. S2). For each mother plant, the expected number of descendants was calculated by multiplying the relative fitness estimate ωesti by the experimental population size (*n*=49) and rounding to the nearest integer.

To construct the DPs, seeds harvested from the four PPs were sown in December 2014 in seedling trays containing compost. The number of seeds sown from each mother plant was equal to the expected number of descendants calculated as explained above, multiplied by 6: that is, the number of descendants was multiplied by 3 to take into account the possibility of low germination rates (i.e. the expected germination rate was 33%) and multiplied by 2 to enlarge the sample size of each DP (so that phenotyping could be performed on 98 individuals per DP rather than 49). Seedling trays were placed in a cold room at 4 °C for 1 week and then transferred to a glasshouse for 8 weeks.

### Test of Zn tolerance in descendant populations

To compare the levels of Zn tolerance among DPs derived from PPs exposed to various levels of Zn in soil, a tolerance test was carried out. At a time *T*_0_, considered to be the beginning of the tolerance test, the available DP seedlings were transferred into individual pots containing 1 kg of a peat- and clay-based compost (70%)/zeolite (30%) mix, contaminated with 500 Zn kg^–1^ by adding 20 ml of a solution of 0.38 M ZnSO_4_·7 H_2_O). This moderate level of Zn exposure was chosen for two main reasons. First, Zn was expected to provoke toxicity but not plant mortality, which would have prevented phenotyping. We made use of the results from the culture of PPs in mesocosms, in which mortality significantly increased from 1000 mg kg^−1^ of Zn exposure. Second, plants had to be healthy enough to actively control metal homeostasis, in particular Zn accumulation, because measuring metal accumulation in weakened plants may give spurious results and lead to erroneous interpretations (Van der Ent *et al.*, 2013).

Pots were randomized and placed in a greenhouse on a testing table. To limit microenvironmental effects, the pots were rotated twice a week. Several traits were recorded at *T*_0_ and after 2 months (*T*_2_). At *T*_0_ and *T*_2_, the number of leaves and the plant surface area (calculated as the surface area of an ellipse from the two larger diameters) were measured in order to calculate a growth rate based on the number of leaves (NL_GR_) and the plant surface (PS_GR_). At *T*_2_, the level of chlorosis was estimated by visually classifying plants into four categories (1, healthy; 2, partially chlorotic; 3, entirely chlorotic; 4, dead plant). Average chlorophyll contents were also measured from three leaves per plant by using a CL-01 Chlorophyll Content Meter (Hansatech Instruments, King’s Lynn, UK). At *T*_2_, a number of biomarkers were analyzed to study the impact of Zn in plant tissues on different physiological processes. These biomarkers were related to (i) levels of pigments and secondary compounds (chlorophyll *a*/*b*, carotenoids, phenolic compounds, flavonoids, and tannins) and (ii) antioxidant enzyme activity [superoxide dismutase (SOD) and ascorbate peroxidase (APX)]. Each biomarker was measured according to the method detailed by Al Souki (2017). Zn concentrations in aerial parts were also measured in three mature leaves following the zincon method developed for *Arabidopsis halleri* (Macnair and Smirnoff, 1999). This method is based on UV-visible spectrophotometry using zincon as a colored Zn-chelating agent and has been previously validated for *N. caerulescens* (Frérot *et al.*, 2005)

### Statistical analyses

For data obtained on PPs in mesocosms, a χ^2^ test was used to compare survival rates among parent populations; pairwise tests were also performed with Bonferroni-adjusted *P*-values. The rates of plants that produced seeds were also compared among PPs using a χ^2^ test and pairwise tests with Bonferroni-adjusted *P*-values.

A principal component analysis (PCA) was performed in order to test whether some traits could contribute to some of the point cloud structure according to zinc contamination levels in PPs. Kruskal–Wallis comparison tests were also performed on each vegetative and reproductive variable to investigate the potential effect of Zn exposure on the plants. This non-parametric test was used because the conditions for ANOVA were not fulfilled. When significant differences were found, *post hoc* Conover tests were performed with Benjamini–Hochberg adjusted *P*-values.

Data obtained on DPs in controlled conditions were used to test the effect of Zn exposure of the PPs in mesocosms on the evolution of metal-related traits. As above, a PCA was performed to test whether some traits could contribute to some of the point cloud structure among DPs. As an ordinal variable, the level of chlorosis was not included in the PCA. Non-parametric Kruskal–Wallis tests were performed for all the continuous variables and for chlorosis. If significant differences were found, *post hoc* Conover tests were performed with Benjamini–Hochberg adjusted *P*-values. Finally, to test whether Zn contamination levels could have modified phenotypic correlations among traits, correlation matrices for each DP were computed and compared using Steiger’s tests.

Comparison tests and correlations were performed with R 3.3.2 (R Core Team, 2016); PCA and graphical representations required the installation of the packages factoMineR and ggplot2.

## Results

### Genotyping of parent populations

None of the pairwise differentiation indices (*F*_ST_) was significant (Supplementary Table S2), indicating strong genetic homogeneity among the four parent populations on the basis of 12 microsatellite markers.

### Comparison of plant performance according to Zn exposure in parent populations

**Fig. 1.**
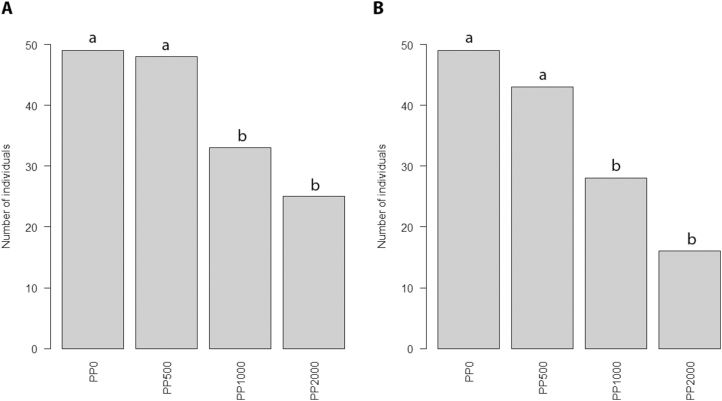
Number of individual plants that (A) survived and (B) produced seeds in each parent population (PP). Bars with different letters are significantly different (*P*≤0.05).

The level of Zn exposure significantly affected survival rates in PPs (χ^2^=50.92, *df*=3, *P*=5.088 × 10^–11^; Fig. 1A): as the contamination level increased, survival rates decreased (PP_0_=100%, PP_500_=98%, PP_1000_=67.3%, PP_2000_=51%). Pairwise tests distinguished two pairs of groups showing significant differences: PP_0_/PP_500_ and PP_1000_/PP_2000_. The number of plants producing seeds decreased as the Zn contamination level increased (PP_0_=100%, PP_500_=87.7%, PP_1000_=57.1%, PP_2000_=32.6%); a χ^2^ test showed significant differences among PPs (χ^2^=63.988, *df*=3, *P*=8.256 × 10^–14^; Fig. 1B).

For surviving plants, the projection of individuals on the two first components of the PCA on vegetative and reproductive traits revealed a slight separation of point clouds, in particular between PP_0_ and PP_2000_ (Supplementary Fig. S3A). In particular, individuals from PP_0_ displayed among the highest coordinates on the first component and the lowest on the second component, whereas individuals from PP_2000_ displayed among the lowest coordinates on the first component and the highest on the second component. The first component explained 49.9% of the variance and combined all reproductive traits, whereas the second component explained 22.6% of the variance and combined vegetative and reproductive traits (Supplementary Fig. S3B, C). Conversely, individuals from PP_500_ and PP_1000_ showed great phenotypic variability. with confidence ellipses largely overlapping the others, either on the first component (for PP_1000_) or on the second component (for PP_500_).

**Fig. 2.**
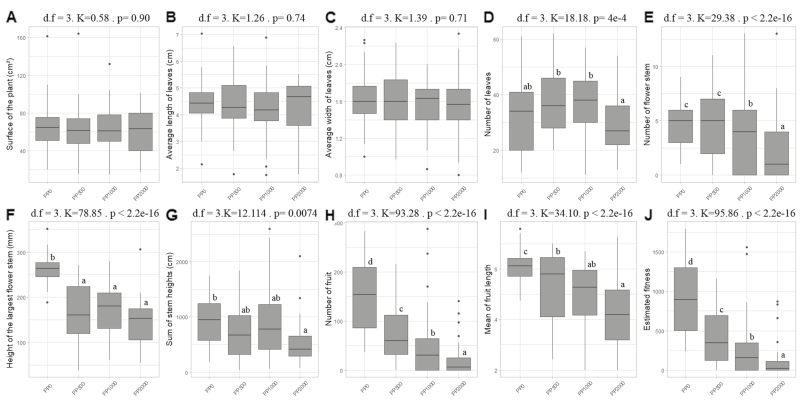
Effect of zinc on vegetative traits (A–D), reproductive traits (E–I), and fitness estimation (J) for each parental population (PP). K, Kruskal–Wallis statistic. Boxplots with different letters are significantly different (*P*≤0.05).

**Fig. 3.**
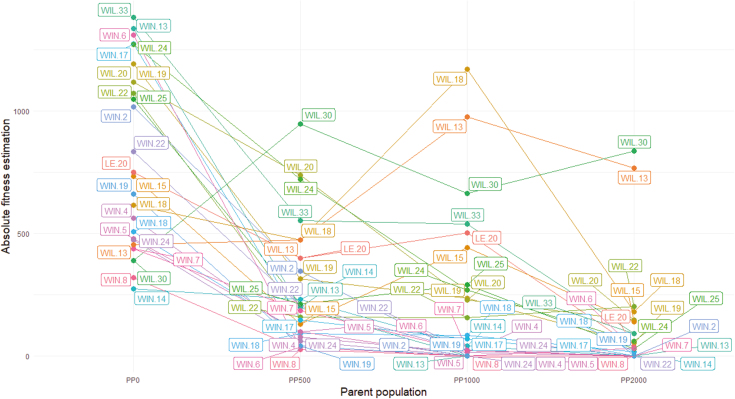
Average absolute fitness estimation by plant family per parent population. LE, Lellingen population; PP, parent population; WIL, Wilwerwiltz population; WIN, Winseler population.

Kruskall–Wallis and *post hoc* tests performed on each variable showed no difference among PPs in terms of the plant surface (Fig. 2A) or the average length and width of the three longest leaves (Fig. 2B, C). Differences among PPs on vegetative traits were apparent only for leaf number (Fig. 2D), with PP_2000_ showing the lowest leaf number (Supplementary Table S3). By contrast, reproductive traits always distinguished PPs, with a reduction of trait values with increasing Zn exposure (Fig. 2E–I). Reproductive outputs were strongly reduced by Zn exposure, with more than a 4-fold reduction in these outputs in PP_2000_ compared with PP_0_ (Fig. 2J). Interestingly, the plants that maintained the best reproductive outputs in PP_500_, PP_1000_, and PP_2000_ belonged to the same families, such as WIL 30, WIL 13, WIL 18, and WIL 24 (Fig. 3).

### Comparison of Zn tolerance levels among descendant populations

**Fig. 4.**
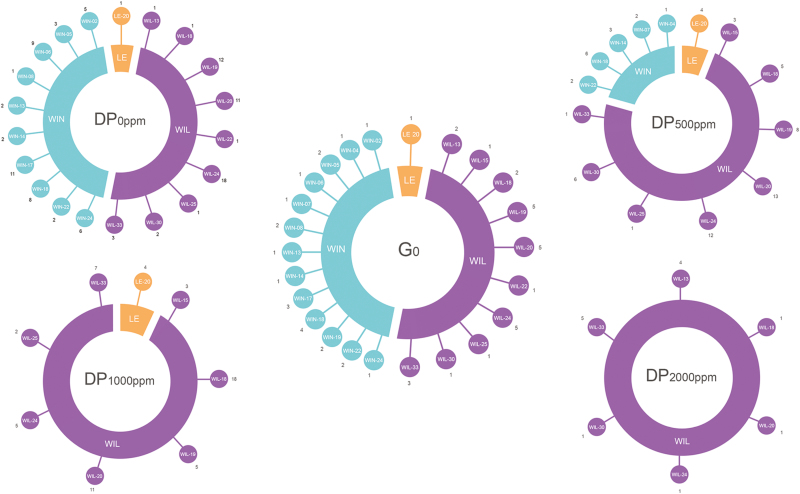
Observed composition of each descendant population (DP_0_, DP_500_, DP_1000_, and DP_2000_) according to the relative fitness of individual mother plants from the corresponding parent populations. LE, Lellingen population; PP, general composition of each parent population; WIL, Wilwerwiltz population; WIN, Winseler population.

Because of poor germination rates, especially for DP_1000_ and DP_2000_, the composition of DPs did not correspond to the expectations from the fitness estimates (Supplementary Fig. S2). In particular, some progenies were represented less than they were expected to be from estimated fitness values, or not at all. Therefore, the composition of the DPs included all available seedlings (Fig. 4).

#### Chlorosis

**Fig. 5.**
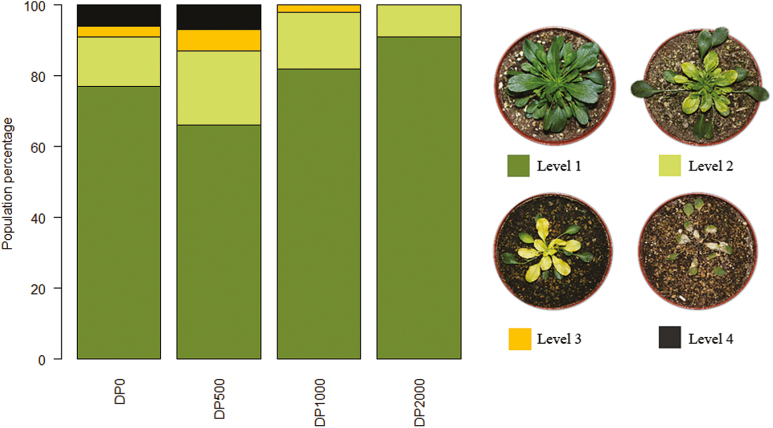
Percentage of plants in each of the four descendant populations (DPs) with each level of chlorosis. Level 1, healthy; level 2, partially chlorotic; level 3, entirely chlorotic; level 4, dead.

At *T*_2_, chlorosis at level 4 (mortality) was observed only in DP_0_ and DP_500_ (Fig. 5). Level 3 chlorosis (plant entirely chlorotic) was observed in DP_0_, DP_500_, and DP_1000_. In DP_2000_, chlorosis was only partial (level 2) when present. However, no significant difference in chlorosis levels was detected between the DPs (χ^2^=4.047, *df*=3, *P*=0.2578).

The PCA performed on continuous data showed that no clear point cloud structure was visible on both axes (Supplementary Fig. S4A). Photosynthetic pigments were mainly represented on the first component (30.9% of variance), while secondary compounds and growth rates were mainly represented on the second component (11.2% of variance) (Supplementary Fig. S4B).

#### Antioxidant enzymes

**Fig. 6.**
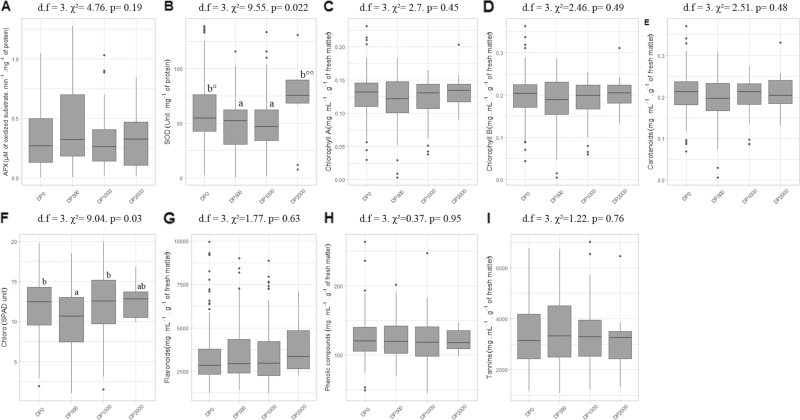
Comparison of biomarker values (activity/concentration) in descendant populations (DPs). (A) Ascorbate peroxidase (APX). (B) Superoxide dismutase (SOD). (C) Chlorophyll *a*. (D) Chlorophyll *b*. (E) Carotenoids. (F) Level of chlorophyll as measured with a chlorophyll meter. (G) Flavonoids. (H) Phenolic compounds. (I) Tannins. Boxplots with different letters are significantly different (*P*≤0.05).

APX activity showed no significant differences among DPs (Fig. 6A). In contrast, significant differences in SOD activity were found among DPs (Fig. 6B). However, this result was not confirmed by Conover’s tests, although DP_500_ and DP_1000_ tended to have significantly lower SOD levels.

#### Photosynthetic pigments and secondary compounds.

Overall, there were no significant differences in concentrations of pigments (chlorophyll *a*, chlorophyll *b*, and carotenoids) among DPs, although DP_500_ showed a tendency towards lower values (Fig. 6C–E). By contrast, chlorophyll content measured with a chlorophyll meter showed significant differences (Fig. 6E). This result was confirmed by Conover’s tests, showing significantly lower chlorophyll contents in DP_500_ than in DP_0_ and DP_1000_. There were no significant differences in the levels of secondary compounds among DPs (Fig. 6F–I).

#### Growth rates

**Fig. 7.**
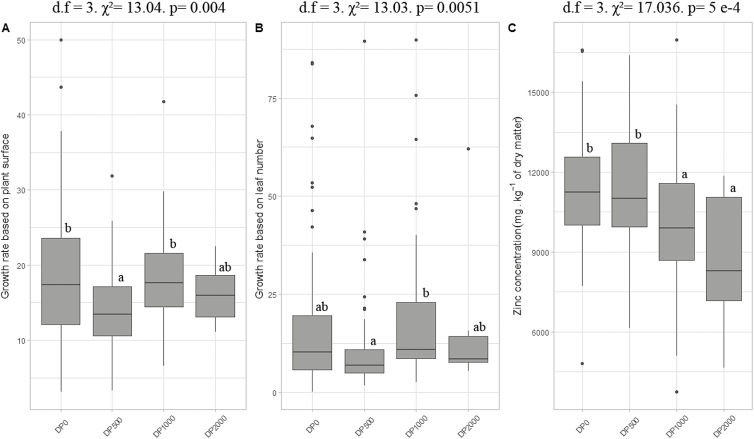
Comparison of growth rates based on (A) surface of the plant or (B) leaf number and (C) zinc concentrations in descendant populations (DPs). Boxplots with different letters are significantly different (*P*≤0.05).

For growth rates measured on leaf number and plant surface, DP_500_ had significantly lower values than DP_0_ and DP_1000_ for SP_Gr_ (Fig. 7A), and a significantly lower value than DP_1000_ for NL_Gr_ (Fig. 7B).

#### Shoot zinc concentrations

Kruskal–Wallis tests showed significant differences among DPs (χ^2^=17.036, df=3, *P*=5 × 10^–04^). Plants from DP_1000_ and DP_2000_ had significantly lower mean ±SD Zn concentrations (DP_1000_, 9896 ± 2407 mg kg^−1^ DW; DP_2000_, 9068 ± 2566 mg kg^−1^ DW) than DP_0_ and DP_500_ (DP_0_, 11433 ± 2182 mg kg^−1^ DW; DP_500_, 10947 ± 2231 mg kg^−1^ DW) (Fig. 7C).

#### Descendant population correlation matrix comparisons

**Fig. 8.**
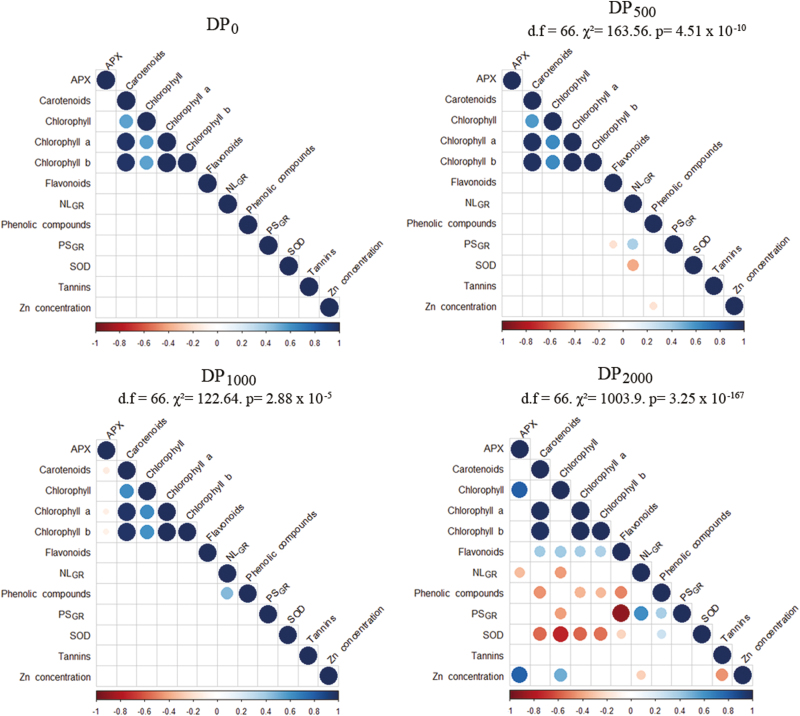
Correlation matrices among traits in descendant populations (DPs). The gradient of colors represents the sign and strength of the correlation. Steiger’s tests indicate comparisons between the DP_0_ correlogram and those of the other DPs. APX, ascorbate peroxidase activity; Carotenoids, concentration of carotenoids; Chlorophyll, Chlorophyll level as measured with a chlorophyll meter; Chlorophyll *a*, chlorophyll *a* concentration; Chlorophyll *b*, chlorophyll *b* concentration; Flavonoids, concentration of flavonoids; NL_GR_, growth rate based on leaf number; Phenolic compounds, concentration of phenolic compounds; PS_GR_, growth rate based on plant surface area; SOD, superoxide dismutase activity; Tannins, concentration of tannins; Zn concentration, shoot zinc concentration.

All correlation matrices showed the same significant positive correlations among photosynthetic pigments. The main differences among matrices came from specific significant negative correlations among other traits. DP_500_ and DP_1000_ were significantly different from DP_0_ on the basis of only a few negative correlations (Fig. 8). Striking differences were evident between DP_0_ and DP_2000_, since several specific correlations could be observed in DP_2000_. Thus, SOD activity was negatively correlated with photosynthetic pigments. Photosynthetic pigments and flavonoid concentrations were also negatively correlated with phenolic compounds and growth rates. In addition, in DP_2000_ , photosynthetic pigments and flavonoid concentrations were positively correlated, as were phenolic compounds and NL_GR_ with PS_GR_.

## Discussion

### Experimental selection for zinc tolerance

In our selection experiment, increasing the level of Zn contamination of the substrate from 0 to 2000 mg kg^−1^ significantly decreased the percentage of plants that survived and produced seeds (Fig. 1). We observed a corresponding strong response of plant reproductive traits, with a progressive reduction of mean values with increasing Zn contamination (Fig. 2). In contrast, except for leaf number, we found no significant difference among PPs for vegetative traits (Fig. 2, Supplementary Fig. S3, Supplementary Table S3). Interestingly, when looking at the identity of families that were able to survive, produce seeds, and maintain a certain level of performance, the effect of increasing the level of Zn contamination in mesocosms was not random. Indeed, the loss of families was apparently gradual, so that most of the families that produced seeds in PP_2000_ also did so in the other conditions. In particular, there was a gradual loss of families from the WIN site with increasing Zn contamination. Of the 13 WIN families initially represented, 13, 12, 2, and 2 produced seeds in PP_0_, PP_500_, PP_1000_, and PP_2000_, respectively. In comparison, of the 10 WIL families initially represented, 10, 10, 10, and 9 produced seeds in PP_0_, PP_500_, PP_1000_, and PP_2000_, respectively. The same pattern was also observed after ranking seed-producing families in terms of their estimated fitness (Fig. 3). The same families, mostly from WIL (e.g. WIL 13, WIL 18, and WIL 30) showed the highest fitness estimates in contaminated culture conditions. As a result, the expected composition of DPs was notably biased towards an overrepresentation of the same few WIL families (Fig. 4). By contrast, WIN families were not represented at all for the phenotyping of PP_1000_ and PP_2000._

The fact that the same families were selected suggests a genetic component in the ability of plants to handle Zn exposure. Since Zn stress mostly affects survival and reproductive traits, it can be considered to be a selective agent influencing the ability of plants to have their genes represented in the next generation (i.e. individual fitness). Any difference among DPs could therefore be interpreted as genetically determined. An alternative explanation would assume that altered phenotypes in DPs may result from transgenerational epigenetic mechanisms, also known as maternal effects, through a form of transgenerational stress adaption (Herman and Sultan, 2011; Weinhold, 2018). This notion remains highly controversial, however, as there is no strong evidence yet that modifications in the DNA methylome induced by environmental stresses can be inherited (Van Dooren *et al.*, 2018, Preprint).

It is clear that our results are limited to the reductionist approach we developed. In either geogenic or anthropogenic soil contamination, Zn never occurs alone, but is usually associated with a cocktail of pollutants as well as other abiotic or biotic modifications of the habitat (Frérot *et al.* 2018). At the organism level, it is known that physiological adaptations may or may not differ when plants are exposed to single or combined stresses (Pandey *et al.*, 2015; Zhang and Sonnewald, 2017). At the genetic level, we can expect that Zn, in combination with other stresses, or in a different chemical form, would affect a certain plant genotype in a different way, or at a different level of exposure.

### Consequences of experimental selection on the evolution of metal tolerance

In our experiments, selection resulting from Zn exposure should have favored evolution towards higher Zn tolerance levels in DPs. Metal tolerance is, however, a very integrative trait that is expected to be determined by a combination of various functional (morphological, physiological, and phenological) traits that operate at lower levels of biological organization and are potentially correlated (Violle *et al.*, 2007).

The traits that we measured in this study included many traits that have been previously considered to be reliable estimates of metal tolerance in plants. Results were, however, not consistent across traits. For some traits, including levels of pigments and secondary compounds as well as APX activity, no difference was observed among DPs. For other traits, a clear trend towards phenotypic divergence among DPs was observed. A trend towards less pronounced chlorosis in the offspring of the PPs exposed to higher levels of Zn, with no entirely chlorotic plants in DP_2000_ only, was observed. DP_2000_ also showed a strong trend towards significantly higher SOD activity compared with other DPs (Fig. 6B). Increased SOD activity also appeared to be associated with new correlations among traits in DP_2000_ that were absent in the other DPs (Fig. 8). This suggests that SOD activity might not have been the only target of selection.

The absence of strong responses raises the question of whether Zn tolerance actually evolved between the PP and DP or whether evolution of Zn tolerance was properly assessed. Two methodological choices may explain the absence of a strong response. First, because we needed non-senescent vegetative plant parts to assess biomarker and metal levels, and because sampling could have biased reproductive traits, we focused on vegetative traits. However, reproductive traits could have revealed larger differences among DPs because they may be closer to fitness (Violle *et al.*, 2007) and therefore to tolerance. Significant differences in metal tolerance among *N. caerulescens* accessions have already been assumed using reproductive traits, while vegetative traits showed no significant difference (Dechamps *et al.*, 2007; Jiménez-Ambriz *et al.*, 2007). Second, exposure of DPs to a single Zn concentration may not have allowed the generation of different responses among DPs. The level of exposure of the DPs (500 ppm) may not have been high enough, or the absence of a control condition (0 ppm), which would have allowed the calculation of tolerance indices, may have limited our ability to detect differences in Zn tolerance among the DPs. The tolerance index statistic was initially designed using root growth, and its relevance to other traits may not necessarily be straightforward. Furthermore, the use of tolerance indices has some other disadvantages that could not be overcome in our study, including the necessity to clone genotypes in a non-clonal species and the need for a large sample size to limit the error variance of the ratio (Macnair, 1993). Finally, statistical analyses may have failed to detect differences between DPs because the sample sizes were unbalanced as a result of contrasting germination and survival rates (N_DP0_=98, N_DP500_=68, N_DP1000_=55, N_DP2000_=11).

In contrast, positive results can be related to previous knowledge. Chlorosis is generally expected to reflect Zn phytotoxicity (Chaney, 1993; Rout and Das, 2003). The absence of chlorosis at most levels of Zn exposure in hydroponic tests has previously been used to show higher Zn tolerance levels of metallicolous compared with non-metallicolous populations of *N. caerulescens* (Assunção *et al.*, 2006). Higher SOD activity can be assumed to reinforce the antioxidant defense system (Sharma and Dietz, 2009). It may help to prevent the damage caused to nucleic acids or photosynthetic pigments by reactive oxygen species produced because of the toxicity of excess internal Zn (Sytar *et al.*, 2013). Increased enzymatic antioxidant capacity may be one of the mechanisms responsible for increased metal tolerance in metal accumulators (Lin and Aarts, 2012). In *N. caerulescens*, it has been suggested that the enhanced activity of antioxidant enzymes such as SOD may result in less accumulation of reactive oxygen species due to Cd toxicity, indirectly resulting in increased Cd tolerance (Wang *et al.*, 2008).

### Consequences of experimental selection on metal hyperaccumulation

Interestingly, our results showed a significantly lower level of Zn hyperaccumulation in the offspring of the PPs that were exposed to the highest levels of Zn (1000 and 2000 mg kg^−1^) compared with the other conditions (0 and 500 mg kg^−1^) (Fig. 5). This may explain the lower levels of shoot chlorosis in DP_1000_ and DP_2000_, since a reduction of the metal burden in the shoots may correspond to reduced metal toxicity (Schat *et al.*, 2000). The lower Zn hyperaccumulation in DP_1000_ and DP_2000_ suggests that selection for increased tolerance could provoke counter-selection for accumulation capacity. A negative relationship between Zn tolerance and Zn accumulation has previously been reported at the population level in *N. caerulescens* (Meerts and van Isacker, 1997). Some degree of negative genetic correlation between these traits was also previously evidenced from cosegregation studies (Assunção *et al.*, 2003*c*; Frérot *et al.*, 2005). So far, however, the evolutionary relationships between metal tolerance and hyperaccumulation remain elusive. Baker (1981) suggested that metal hyperaccumulation was one of the two physiological strategies of metal tolerance and that it could have been selected to increase metal tolerance because the active concentration of metals in deciduous aerial parts of plants could allow metals to be released into the external environment and therefore reduce the overall metal load. Alternative, not mutually exclusive, hypotheses have also been formulated (Boyd and Martens, 1992). The hypothesis of drought resistance assumes that metal hyperaccumulation could have been selected to increase cellular osmolarity in dry environments. The hypothesis of elemental allelopathy assumes that hyperaccumulation of metal may be useful against nearby competitors (Boyd and Martens, 1992; Boyd, 1998). The elemental defense hypothesis, which Vesk and Reichman (2009) described as ‘the leading hypothesis for the evolution of the metal hyperaccumulation trait in plants’, assumes that the accumulation of metals could be advantageous because it renders plants toxic for herbivores or pathogens (Boyd and Martens, 1992; Boyd, 1998, 2007). A recent development of this hypothesis even suggests that hyperaccumulation could be stimulated by wounding (Plaza *et al.*, 2015). Another hypothesis assumes that metal hyperaccumulation could have evolved accidentally, as a by-product of other physiological modifications; if this were the case, it would therefore be non-functional, non-advantageous, and would have no impact on plant fitness. Apart from suggesting that hyperaccumulation may evolve under selection pressure, our results do not invalidate any of these other hypotheses. They suggest that, in species showing species-wide Zn hyperaccumulation, selection towards higher Zn tolerance levels may show a trade-off with evolution towards higher hyperaccumulation levels under conditions of exposure to high Zn concentrations. This may correspond to a reduction of the metal burden in the shoot when metal concentrations in soils are high.

## Conclusion

Our selection experiment suggested that high Zn contamination affected several fitness components of non-metallicolous individuals of *N. caerulescens*, such as survival, reproductive traits, and seed production. After one generation of selection, a substantial response appeared at the highest Zn contamination levels. In particular, a trend towards more pronounced physiological traits associated with Zn tolerance, such as chlorophyll level and SOD activity, was observed. Our results also suggest that selection towards increased Zn tolerance could be associated with a reduction of Zn hyperaccumulation capacity. These results therefore support a role of Zn contamination as a possible selective agent shaping the evolution of both Zn tolerance and Zn hyperaccumulation. Results will have to be confirmed in further studies, in particular to check whether Zn contamination still represents a selective pressure in the field. If our results are confirmed, the molecular bases of adaptive phenotypic divergence among metallicolous and non-metallicolous populations could also be investigated.

## Supplementary data

Supplementary data are available at *JXB* online.

Fig. S1. Lysimeter schema.

Fig. S2. Expected composition of each descendant population.

Fig. S3. Principal components analysis results on PP data.

Fig. S4. Principal components analysis results on DP data at T_2_.

Table S1. Population composition of parent populations.

Table S2. Pairwise genetic differentiation indices (F_ST_).

Table S3. Results of comparison tests on traits measured in parent populations.

Supplementary Tables and Figures
