# Supplementary material for: Can zinc pollution promote adaptive evolution in plants? Insights from a one-generation selection experiment
Source: J Exp Bot. 2018 Sep 12;69(22):5561–72. doi: 10.1093/jxb/ery327 (PMC6255711; doi:10.1093/jxb/ery327)
Supplement: Supplementary Tables and Figures [file ery327_suppl_supplementary_tables_figures.pdf]

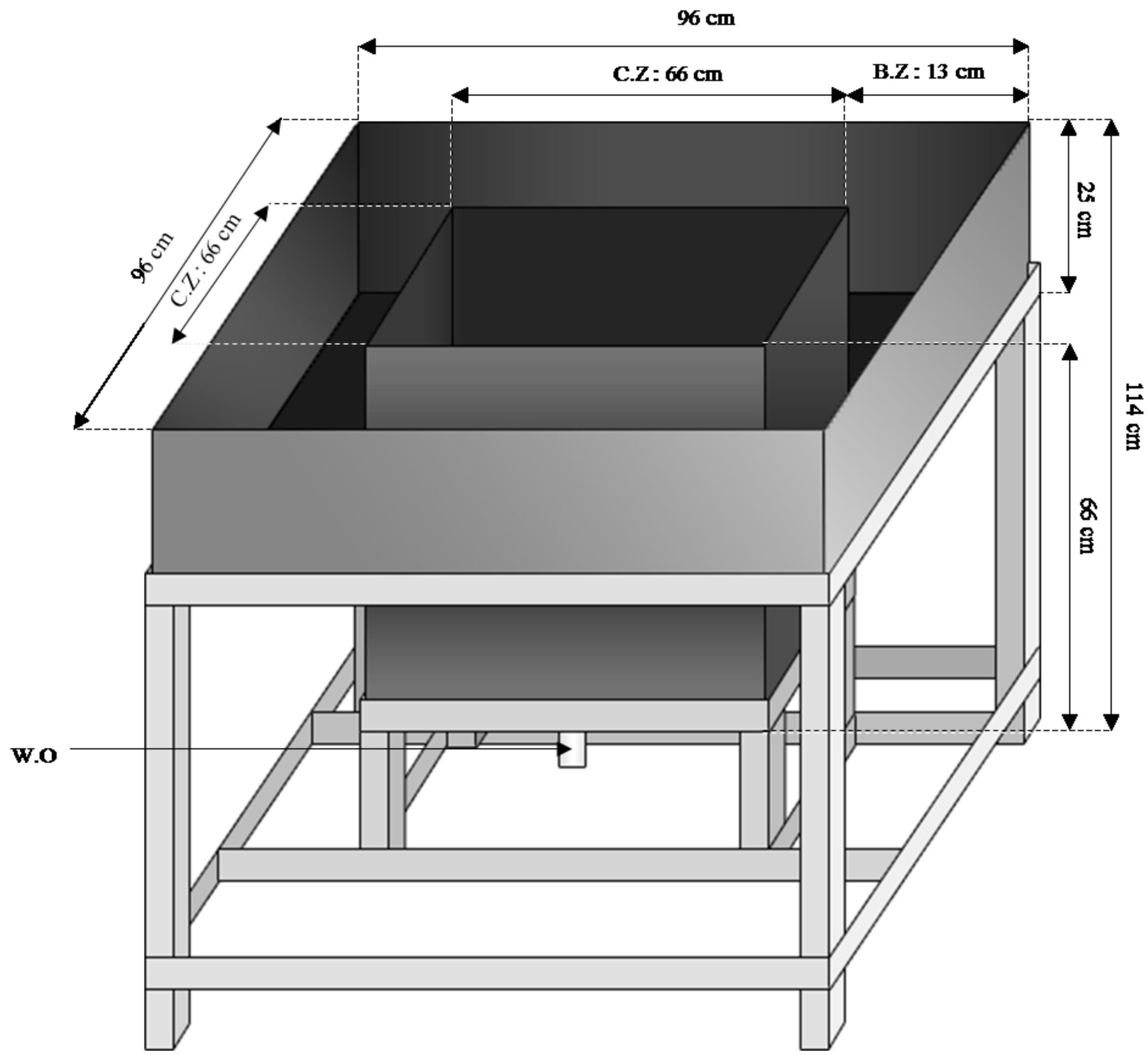

Figure S1: Lysimeter schema. B.Z: Buffer zone. C.Z: Cultivation zone. W.O: Water outlet

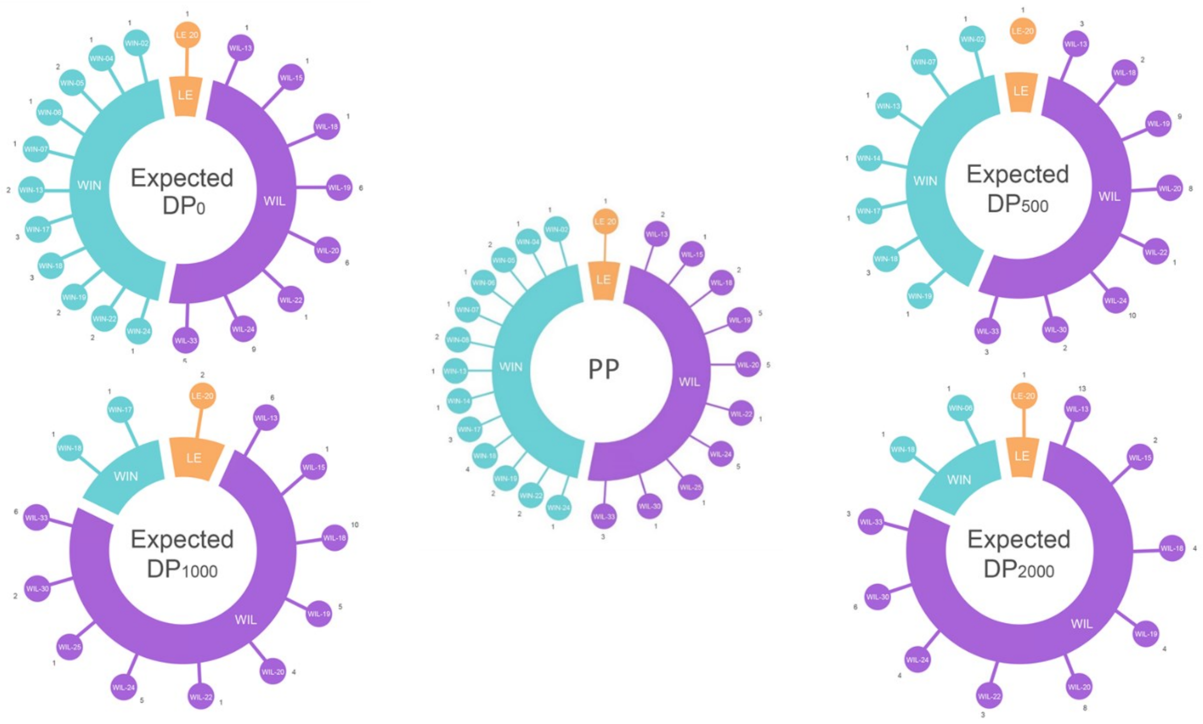

Figure S2: Expected composition of each descendant population (DP_0, DP_{500}, DP_{1000}, DP_{2000}) according to the relative fitness of individual mother plants from the corresponding parent populations. PP: general composition of each parent population; WIN: Winseler population; WIL: Wilwerwiltz population; LE: Lellingen population.

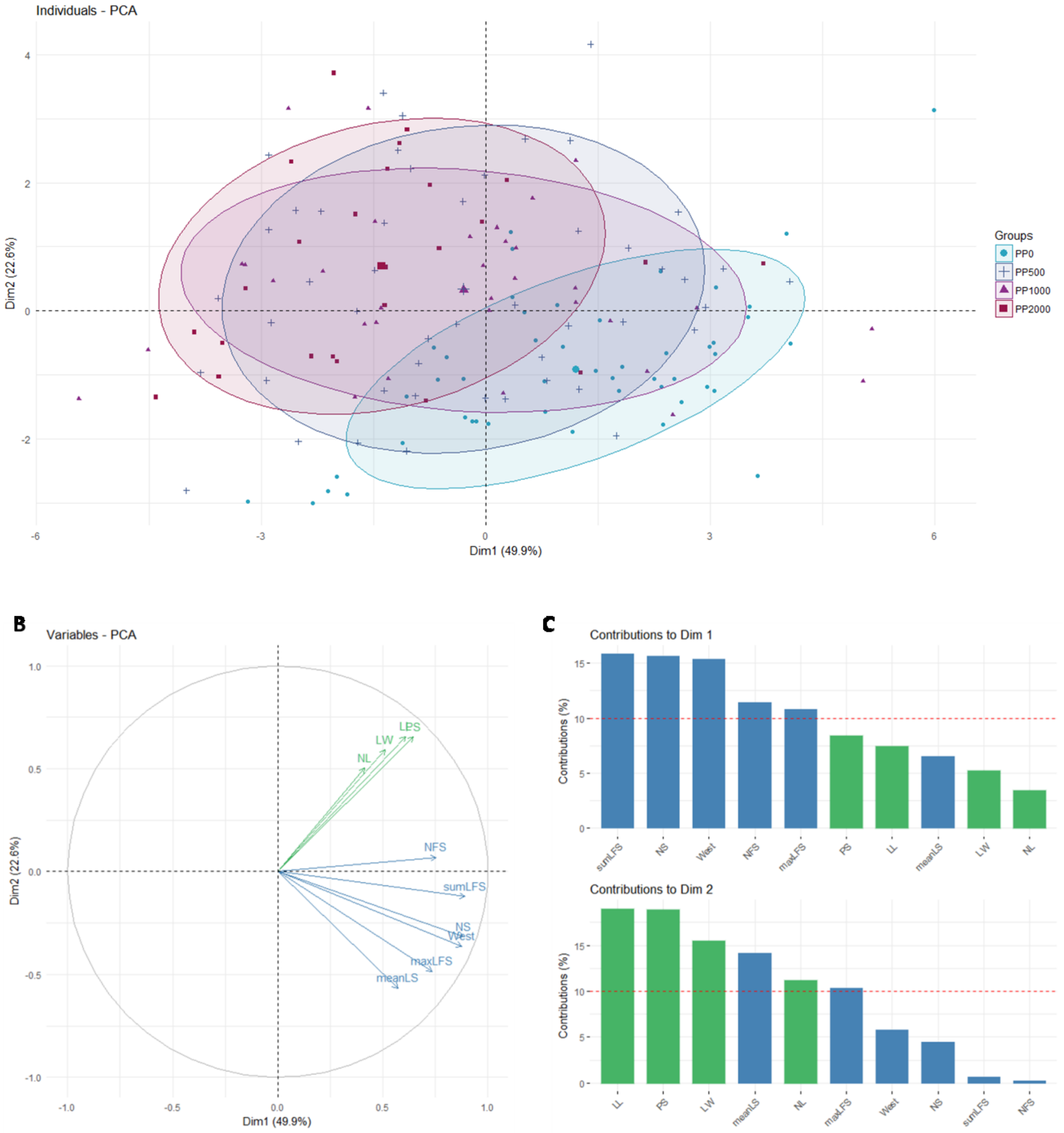

Figure S3: Principal components analysis results on PP data. (A) Projection of individuals. (B) Correlation circle. (C) Variable contribution in each dimension showing vegetative (green bars) and reproductive (blue bars) traits. PP: parent population; PS: plant surface; NL: number of leaves; LL: leaf length; LW: leaf width; NFS: number of flower stems; maxLFS: the length of the longest stem; sumLFS: the sum of lengths of flower stems; NS: the total number of non-aborted siliques; meanLS: the mean length of silique for the entire plant; West: estimated absolute fitness.

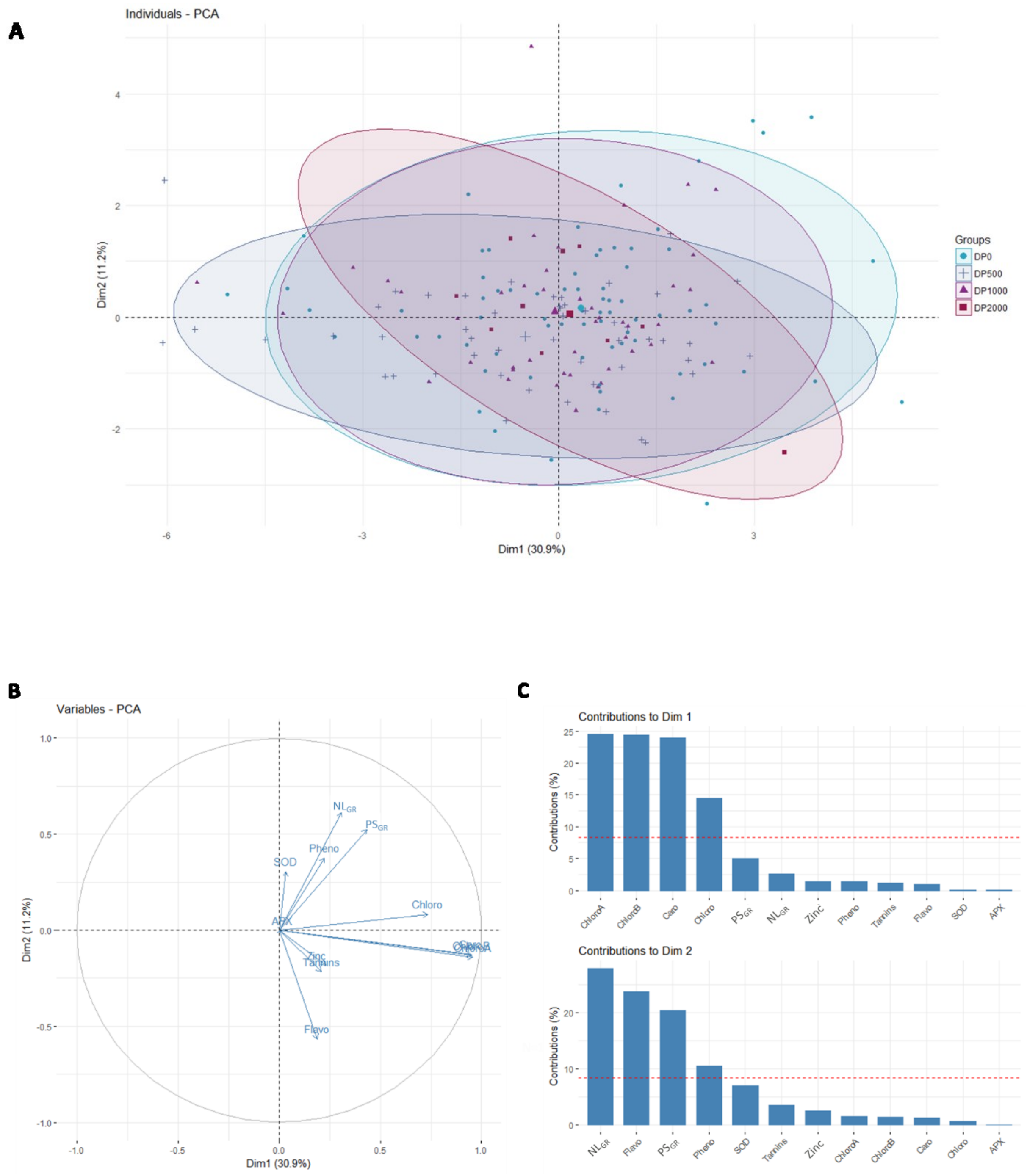

Figure S4: Principal components analysis results on DP data at T_2. (A) Projection of individuals. (B) Correlation circle. (C) Variable contribution in each dimension. DP: parent population; ChloroA: leaf concentration in chlorophyll a; ChloroB: leaf concentration in chlorophyll b; Chloro: chlorophyll rate measured with a Chlorophyll Meter; Zinc: leaf zinc concentration; Caro: leaf concentration in carotenoids; Flavo: leaf concentration in phenolic compounds; Tannins: leaf concentration in tannins; APX: activity of the ascorbate peroxidase enzyme; SOD: activity of the superoxide dismutase enzyme; NL_{GR}: growth rate based on leaves number; PS_{GR}: growth rate based on plant surface.

Table S1: Population composition of parent populations represented by the plant position in each mesocosm on a 7*7 grid. Each name corresponds to the name of the mother plant. Each mesocosm received a descendant from the same mother plant.

	**1**	**2**	**3**	**4**	**5**	**6**	**7**
**1**	WIN-18	WIL-18	WIL-19	WIN-14	WIN-04	WIN-05	WIN-08
**2**	WIN-22	WIL-22	WIL-20	WIL-15	WIN-13	WIL-33	WIN-24
**3**	WIN-07	WIN-17	WIL-25	LE-20	WIL-13	WIN-02	WIN-06
**4**	WIL-24	WIN-19	WIL-30	WIN-18	WIL-18	WIL-19	WIN-05
**5**	WIN-08	WIN-22	WIL-20	WIL-33	WIN-17	WIL-13	WIL-24
**6**	WIN-19	WIN-18	WIL-19	WIL-20	WIL-33	WIN-17	WIL-24
**7**	WIN-18	WIL-19	WIL-20	WIL-24	WIL-19	WIL-20	WIL-24

Table S2: Pairwise genetic differentiation indices (F_{ST}) calculated from the polymorphisms of 12 microsatellite markers among the four parental populations (PPs). None of the F_{ST} values was significant.

	**PP_0**	**PP_{500}**	**PP_{1000}**	**PP_{2000}**
**PP_0**	-	-0.0162	-0.0169	-0.0142
**PP_{500}**	-0.0162	-	-0.0177	-0.0151
**PP_{1000}**	-0.0169	-0.0177	-	-0.0138
**PP_{2000}**	-0.0142	-0.0151	-0.0138	-

Table S3: Results of comparison tests on traits measured in parent populations. K= Kruskal-Wallis statistic. d.f= degree of freedom. PP: parent population. Different letters show significant differences at the 5% level for the *post-hoc* Conover's test. Green lines indicate vegetative traits. Blue lines indicate reproductive traits.

Traits	K	d.f	p.value	mean ± sd (PP_0)	mean ± sd (PP_{500})	mean ± sd (PP_{1000})	mean ± sd (PP_{2000})
Plant Surface	0.58	3	0.90	64.45 ± 23.6	63.32 ± 24.75	63.94 ± 23.96	60.31 ± 23.15
Leaf lenght	1.26	3	0.74	4.41 ± 0.83	4.35 ± 0.93	4.21 ± 0.96	4.3 ± 0.93
Leaf width	1.39	3	0.71	1.64 ± 0.3	1.64 ± 0.33	1.57 ± 0.27	1.57 ± 0.32
Number of leaves	18.18	3	$4e^{-4}$	32.92 ± 12.04^{ab}	37.08 ± 10.7^{b}	37.04 ± 10.64^{b}	28.86 ± 10.35^{a}
Number of flower stems	29.38	3	$< 2.2e^{-16}$	4.71 ± 2.08^{bc}	4.96 ± 2.84^{c}	3.96 ± 3.84^{b}	2.27 ± 2.96^{a}
Length of the longest stem	78.85	3	$< 2.2e^{-16}$	263.1 ± 27.45^{b}	169.29 ± 59.13^{a}	172.12 ± 61.9^{a}	147.56 ± 53.35^{a}
Sum of lengths of flower stems	12.114	3	0.0074	903.4 ± 434.7^{b}	706.5 ± 455.1^{ab}	928.7 ± 705.1^{ab}	551.6 ± 454.7^{a}
Number of non-aborted siliques	93.28	3	$< 2.2e^{-16}$	145.94 ± 64.83^{d}	74.12 ± 52.89^{c}	46.16 ± 61.02^{b}	18.73 ± 30.79^{a}
Mean length of siliques	34.10	3	$< 2.2e^{-16}$	6.15 ± 0.64^{c}	5.26 ± 1.42^{b}	4.82 ± 1.47^{ab}	4.24 ± 1.37^{a}
Absolute fitness estimation	95.86	3	$< 2.2e^{-16}$	897.7 ± 408.55^{d}	431.4 ± 354.5^{c}	250.1 ± 351.04^{b}	97.45 ± 196.53^{a}
